# Eliminating manganese sensitivity and boosting citric acid production in *Aspergillus niger* through transporters manipulation

**DOI:** 10.1186/s12934-026-03082-y

**Published:** 2026-08-06

**Authors:** Alexandra Márton, Josee Vandal, Vivien Bíró, Erzsébet Fekete, Erzsébet Sándor, Béla Kovács, Adrian Tsang, Levente Karaffa

**Affiliations:** 1 Department of Biochemical Engineering, Faculty of Science and Technology, University of Debrecen, Debrecen, Hungary; 2 Centre for Structural and Functional Genomics, Concordia University, Montreal, QC Canada; 3 Institute of Plant Protection, Faculty of Agricultural and Food Science and Environmental Management, University of Debrecen, Debrecen, Hungary; 4 Present Address: Department of Molecular Biotechnology and Microbiology, Faculty of Science and Technology, University of Debrecen, Debrecen, Hungary

**Keywords:** *Aspergillus niger*, Citric acid, Manganese(II) ions, Manganese transporter, Citrate transporter, Fermentation

## Abstract

**Background:**

Citric acid, a high-volume bioproduct, is manufactured by submerged fermentation using the filamentous fungus *Aspergillus niger*. Citric acid overflow requires Mn(II) ion concentrations not exceeding 5 µg L^− 1^. However, manganese easily leaches from media ingredients or stainless-steel bioreactors, necessitating costly, laborious removal.

**Results:**

*Aspergillus niger* citrate fermentations were evaluated across varied manganese profiles, comparing a novel double transporter mutant against parental and single-mutant strains. This strain overexpresses *cexA* – the primary plasma membrane exporter whose activity overcomes a major production bottleneck – under the control of the strong *glaA* promoter in a background deficient in *dmtA*, which encodes the primary high-affinity NRAMP-family manganese transporter. Under high-manganese conditions (100–165 µg L^− 1^) simulating industrial substrate impurities and bioreactor leaching, the double transporter mutant outperformed the other strains. Despite abundant manganese that triggers high biomass (50 g L^− 1^) and low citrate yields in the parental strain NRRL2270, this cell factory maintained high-yield overflow metabolism. Driven by an accelerated maximal glucose consumption rate of 1.61 g L^− 1^ h^− 1^ and reduced biomass accumulation (9.1 g L^− 1^), it accumulated citric acid to levels comparable to those achieved under manganese deficiency. The double transporter mutant compressed the fermentation period by 17% (from 300 to 250 h), minimizing operational energy and utility costs and significantly enhancing overall productivity. To validate industrial scalability, the untreated, high-manganese process was replicated in 15-L stainless steel bioreactors – accounting for up to 30 µg L^− 1^ of manganese leaching – and successfully scaled up by an order of magnitude in a 150-L bioreactor. Carbon balance closures within ± 5% confirmed no appreciable by-product formation.

**Conclusions:**

By demonstrating the maintenance of high-yield overflow metabolism under manganese-replete conditions, alongside enhanced productivity through shortened fermentation times across different vessel scales, this study establishes this *A. niger* mutant as an attractive cell factory for industrial applications.

**Supplementary Information:**

The online version contains supplementary material available at https://doi.org/10.1186/s12934-026-03082-y.

## Introduction

Citric acid (2-hydroxypropane-1,2,3-tricarboxylic acid) is the most widely produced organic acid in industrial biotechnology. Owing to its acidity, buffering capacity, metal-chelating properties, and recognized safety, it is extensively used in the food, beverage, pharmaceutical, cosmetic, and detergent industries [1, 2]. Global production of citric acid currently exceeds 2.5 million tons annually, with almost all industrial citric acid being produced by submerged fermentation (usually in batch mode) using the filamentous Ascomycete fungus *Aspergillus niger* [3, 4]. The dominance of *A. niger* as a production platform is attributed to its high capacity for organic acid secretion, tolerance to low pH, efficient utilization of inexpensive carbohydrate substrates, and long history of safe industrial application [3, 5, 6].

Citric acid accumulates through the condensation of oxaloacetate and pyruvate. A distinctive biochemical hallmark of *A. niger* (and a few other fungi) is that the oxaloacetate is formed by fixing carbon dioxide from one of the two glucose-derived pyruvate molecules in the cytosol ([7, 8, 9] while the other undergoes oxidative decarboxylation to acetyl-CoA (Cleland-Johnson reaction, [10]). This pathway prevents carbon dioxide loss, enabling molar yields to exceed the 66% maximum achieved by the standard TCA cycle [11]. Consequently, citrate accumulation is classified as ‘overflow’ and fermentations as ‘high-yield’ when exceeding this 66% threshold – a benchmark adopted in this study.

High-yield citrate fermentation requires elevated initial sugar concentrations (> 120 g L^-1^), low pH (< 1.5), and robust dissolved oxygen (DO) levels, alongside restricted nitrogen, phosphate, and trace metal ion concentrations in the growth media [3, 5, 12]. Particularly critical is the strict limitation of Mn^2+^ ions (hereafter ”manganese”) to concentrations not exceeding 5 µg L^-1^ (= parts per billion, ppb) at the early stages of the process [3, 5, 13–15]. Modulating manganese concentrations acts as a metabolic switch, toggling *A. niger* between citrate overflow and standard growth-oriented pathways through this single parameter even under otherwise optimized conditions [16]. The physiological basis of the ”manganese effect” involves fungal morphology (swollen hyphae and the formation of small, compact pellets), cell wall composition (increased chitin and reduced β-glucan levels), and metabolic regulation [15].

Cellular manganese uptake in *A. niger* is primarily mediated by the high-affinity NRAMP-family transporter DmtA [16]. Deletion of *dmtA* eliminates manganese uptake at low manganese concentrations. Manipulation of DmtA affects citric acid overflow and the associated fungal morphology, linking manganese transport to citrate production physiology.

Another strategy to improve citric acid production involves engineering transport systems responsible for citrate export. The citrate exporter CexA is the primary plasma membrane transporter in *A. niger* [17]. Its disruption halts while overexpression boosts citrate secretion, thereby identifying transport as a production bottleneck. Elevated manganese concentrations suppress *cexA* transcription, directly linking manganese homeostasis to citrate export and providing a mechanistic explanation for the ”manganese effect” [18].

In this study, we demonstrate that an *A. niger* mutant strain, where *cexA* is overexpressed under the control of the strong glucoamylase (*glaA*) promoter in a *dmtA*-deficient background, can accumulate citric acid to levels even higher than those of the parent (i.e., unmutated transporters) strain under manganese-deficient conditions, despite the presence of abundant manganese. Furthermore, the fermentation time – a critical parameter in industrial processes—is significantly reduced, making this strain an attractive cell factory for industrial applications.

## Materials and methods

### Fungal strains and mutant generation

All strains used in this study are listed and described in Table 1.

**Table 1:** *Aspergillus niger* strains used in this study

Name	Parent	Genotype	References
NRRL 2270 (A60; ATCC 11414)	NRRL 328 (ATCC 1015)	Citric acid hyperproducer; Spontaneous derivative	Perlman et al. 1946
CSFG_7003	NRRL2270	*ΔpyrG ΔkusA*	Song et al. [21]
JPO_1	CSFG_7003	*ΔpyrG ΔkusA ΔdmtA* ANEp8-Cas9-gRNAdmtA	Fejes et al. [16]
CSFG_7012_1	CSFG_7003	*ΔpyrG ΔkusA ΔglaA::cexA* ANEp8-Cas9-gRNAglaA	This study
CSFG_7013_1	JPO_1	*ΔpyrG ΔkusA ΔdmtA ΔglaA::cexA* ANEp8-Cas9-gRNAglaA	This study

Overproduction of the citrate exporter in *A. niger* was achieved by replacing the coding region of the glucoamylase gene with the *cexA* gene (ID NRRL3_06530) using the same plasmids and protocol as described by Semper et al. [19]. Briefly, the *cexA* gene was PCR-amplified from *A. niger* NRRL2270 genomic DNA using Phusion DNA polymerase. The PCR product was recombined with pJET, a plasmid containing 655 and 700 bp of the glucoamylase promoter and the terminator regions, by ligation-independent cloning [20]. The resulting plasmid was introduced along with ANEp8-Cas9-gRNAglaA [21] to *A. niger* strains CSFG_7003 and JPO_1 to generate strains CSFG_7012_1 and CSFG_7013_1, respectively. The latter is henceforth referred to as ”double transporter mutant”.

### Media and cultivation conditions

The *A. niger* strains were maintained as conidial spores on agar plates stored at 4 °C. The maintenance medium was adjusted to pH 6 and composed of (per litre) 10 g D-glucose, 6 g NaNO_3_, 1.5 g KH_2_PO_4_, 0.5 g MgSO_4_·7H_2_O and 0.5 g KCl. In addition, 20 µL L^-1^ of a trace element solution was included that contained (per litre) 10 g EDTA, 4.4 g ZnSO_4_·7H_2_O, 1.01 g MnCl_2_·4H_2_O, 0.32 g CoCl_2_·6H_2_O, 0.315 g CuSO_4_·5H_2_O, 0.22 g (NH_4_)_6_Mo7O_24_·4H_2_O, 1.47 g CaCl_2_·7H_2_O and 1.1 g FeSO_4_·7H_2_O.

For inoculum preparation, conidia of *A. niger* were harvested from freshly prepared plates and suspended in an aqueous 0.01% Tween 20 solution to obtain a dense spore suspension. Seed cultures were established by inoculating the growth medium to a final concentration of 5 × 10^6^ conidia mL^-1^. Cultivation was performed in 500-mL Erlenmeyer flasks containing 100 mL medium (VWR International Kft., Debrecen, Hungary). The flasks were incubated for 24 h at 30 °C in a rotary shaker (Infors AG, Basel, Switzerland) operating at 250 rpm. The seed medium contained D-glucose as the sole carbon source at an initial concentration of 10 g L^-1^ and was supplemented with (per litre) 2.50 g (NH_4_)_2_SO_4_, 0.15 g KH_2_PO_4_, 0.15 g NaCl and 2.25 g MgSO_4_·7H_2_O. Trace metals were present at concentrations of 1.50 mg L^-1^ Zn^2+^, 0.10 mg L^-1^ Fe^2+^ and 0.06 mg L^-1^ Cu^2+^. Prior to sterilization, the pH of the medium was adjusted to 3.0 with 3 M HCl. No pH regulation was applied during the subsequent shake-flask cultivation.

Citric acid fermentations were carried out in a chemically defined medium identical to the seed culture formulation except for the higher initial concentration of D-glucose, set to 140 g L^-1^ [16]. To regulate the manganese content, the glucose was dissolved in distilled water and the solution was passed through a Dowex 50 W-X8 (100/200) cation-exchange resin column (440 × 45 mm). The manganese concentration was set by supplementing the medium with MnCl_2_·4H_2_O at either 5 µg L^-1^ or 100 µg L^-1^ (termed ”low manganese” or ”high manganese” conditions, respectively). In the third set of experiments (hereafter ‘untreated’ conditions), tap water was used for medium preparation instead of distilled water, and the glucose solution was not subjected to cation exchange; consequently, the manganese content of the growth medium varied across these experiments, but significantly exceeded the threshold of ≤ 5 µg L^-1^.

Media were sterilized by membrane filtration under aseptic conditions and transferred into previously autoclaved and cooled shake flasks or fermenters. These vessels already contained the required volume of ion-exchanged and subsequently double-distilled water, hereafter referred to as ”manganese-free water”. All chemicals used in the study were of analytical grade and were obtained from Sigma-Aldrich Ltd. (Budapest, Hungary).

Citric acid fermentations were carried out in batch mode in 500 mL Erlenmeyer shake-flasks incubated at 30 °C in a rotary shaker (Infors AG, Basel, Switzerland) at 250 rpm, a pair of 6-L-scale autoclavable glass fermenters (Sartorius Biostat B, Göttingen, Germany), two 15-L scale in situ sterilizable stainless steel fermenters (Zolend Ltd., Debrecen, Hungary), or a 150-L pilot scale stirred tank reactor (Gyémánt-Pirazol Ltd., Debrecen, Hungary; Additional file 1: Fig. S1). The metal components of all bioreactors are made of AISI 316 L (≤ 2% manganese content), the most commonly used grade of stainless steel in biotechnological applications [22]. The bioreactors (6-L, 15-L, and 150-L alike) feature six-blade Rushton-type disc turbine impellers. The 6-L units use two 65 mm impellers; the 15-L units use three impellers (a 85 mm lower unit and two 73 mm upper units); and the 150-L units use three 216 mm impellers.

Bioreactors (6, 15, and 150 L) were sterilized under 1 bar overpressure (~ 121 °C) using scale-specific methods. The 6-L vessels were autoclaved for 30 min containing manganese-free water, then aseptically supplemented with deionized D-glucose and pre-sterilized medium components. The 15-L and 150-L units were sterilized in situ: the former via internal electrical heating elements for 40 min, and the latter by steam injection into the vessel jacket for 60 min.

Scale-up from the 6-L laboratory bioreactor to the 150-L pilot-scale vessel was performed using a constant impeller tip speed criterion (v_t_ = π * N * D_i_/60000, where N is the rotational speed in rpm, D_i_ is the impeller diameter in mm, and v_t_ is the tip speed in m/s) to maintain comparable maximum shear conditions, combined with a constant volumetric aeration rate of 0.75 vessel volume per minute (vvm). To accommodate dynamic changes in the oxygen transfer rate during biomass accumulation, the DO level was continuously maintained at 30% saturation across both scales via automated cascade control loops adjusting the agitation speed.

The initial pH of the growth medium was adjusted to 3.0 with 3 M HCl before inoculation. The pH was measured but not controlled during fermentations. Temperature, DO and impeller tip speed were controlled automatically by the controller units of the bioreactors. To minimize medium loss, the waste gas from the headspace was cooled in a reflux condenser connected to a cooling bath (4 °C) before exiting the system. Fermentations were inoculated under aseptic conditions with harvested and washed biomass from 10% of seed culture, and run until the initial D-glucose of 140 g L^-1^ was depleted, which defined the total fermentation time.

To mitigate and monitor potential manganese leakage from the stainless-steel components of the 6-L glass bioreactors (containing up to 2% manganese [23], vessels were electrochemically polished prior to use, and manganese concentrations were regularly quantified during fermentation. In contrast, the larger-scale (15-L and 150-L) stainless-steel bioreactors were deliberately left unpolished and unmonitored for manganese levels. This setup was designed to evaluate the performance of the double mutant strain under unmitigated, elemental leaching conditions, assessing its industrial viability without the need for cost-intensive manganese monitoring and control.

### Analytical methods

D-glucose and citrate concentrations (the latter also referred to as titer; g L^-1^) in the culture medium were quantified by high-performance liquid chromatography (HPLC; Agilent Technologies 1260 Infinity II, United States). Analyses were performed on a Bio-Rad Aminex HPX-87 H^+^ column maintained at 55 °C, using isocratic elution with 10mM H_2_SO_4_ and refractive index detection [24]. The concentrations were calculated from two independent measurements, which never deviated more than 5%. Maximal productivity (g citrate L^-1^ h^-1^) was calculated from the biggest increase in citrate concentrations between two consecutive samplings. Similarly, maximal glucose consumption rates (g L^-1^ h^-1^) were determined from the sharpest decrease in residual glucose concentrations between two consecutive samplings. Specific molar citric acid yield (Y_p/s_) is the ratio of the produced moles of citric acid and the consumed moles of D-glucose.

Mycelial dry cell weight (DCW) was determined from 10 mL culture aliquots as described by Kozma and Karaffa [25]. Biomass was collected on a pre-weighed glass wool filter, washed with cold tap water and dried at 80 °C to constant weight. Biomass yields (Y_x/s_) were calculated by relating the concentrations of the final biomass (DCW) to that of the total carbon source used. Maximal biomass production rates (g_DCW_ L^-1^ h^-1^) were calculated from the highest increase in DCW between two consecutive sampling points. Similarly, the maximal specific growth rate (µ_max_, h^-1^) during the fermentation was determined as follows: (µ_max_ = [ln(X_2_/X_1_)] / (t_2_ - t_1_), where (X_1_) and (X_2_) represent the DCW concentrations at consecutive sampling times (t_1_) and (t_2_) exhibiting the steepest growth.

The concentration of manganese in the culture broth was quantified by inductively coupled plasma quadrupole mass spectrometry (Thermo Fisher Scientific, Bremen, Germany) equipped with Hexapole Collision Cell Technology [26].

Fungal morphology were monitored by Zeiss AxioImager phase-contrast microscope, equipped with AxioCam MRc5 camera and analysed with an AxioVision AC quantitative image analyser system. Image contrast was improved by adding lactophenol cotton blue (Fluka Chemie, Buch, Switzerland) to a final concentration of 10% (v/v).

### Reproducibility

All data from fungal cultivations represent the mean of three independent biological replicates (using different spore inocula). Each value represents the average of two technical replicates (two parallel measurements within the same experiment). Analysis and visualization were performed using SigmaPlot software (Jandel Scientific, San Jose, CA, United States). Variability was expressed as standard deviation of each dataset. Statistical evaluation of quantitative datasets (*n* ≥ 3) was performed using analysis of variance (ANOVA) followed by the Holm–Sidak post hoc test for pairwise comparisons. Although *p*-values frequently fell below 0.001, a threshold of *p* < 0.05 was applied to determine statistical significance.

## Results and discussion

### Setting up the experimental system

Three parallel sets of 6-L scale citrate fermentations (each performed in triplicate) were conducted under identical, optimized operational conditions, varying in their medium purification levels and corresponding manganese profiles. Fully optimized control fermentations utilizing deionized medium components were maintained at a threshold of ≤ 5 µg L^− 1^ manganese. These were compared against two distinct setups characterized by elevated manganese levels. The first elevated condition introduced a fixed concentration of 100 µg L^− 1^ manganese into a deionized medium—a concentration previously shown to compromise citrate yields by two-thirds [16]. The second elevated configuration simulated an industrially relevant, economically viable scenario by utilizing untreated components as follows. Tap water replaced deionized water and introducing seasonally fluctuating manganese levels between 2.5 and 23.4 µg L^− 1^ (Additional file 2: Table S1). Furthermore, omitting ion-exchange resin purification for the concentrated D-glucose stock led to an additional manganese influx of up to 150 µg L^− 1^ from the carbon source itself [26]. Consequently, initial manganese levels under these untreated conditions ranged from 148 to 165 µg L^− 1^. Although other metal ions and impurities were inevitably introduced through the tap water and unpurified glucose solution, these were not quantified, as the objective of this setup was to evaluate the performance of the double mutant strain in an untreated growth medium subject to compositional variations.

To assess whether elemental leaching from the steel alloy influenced process performance, fermentation with the untreated medium was replicated in 15-L stainless steel bioreactors. Depending on their level of corrosion, such fermenters can introduce up to 30 µg L^− 1^ of manganese into the medium during sterilization and subsequent cultivation at low pH [14]. Finally, to evaluate scalability, the untreated process was scaled up by an order of magnitude in a 150-L bioreactor.

To verify whether the carbon source was channeled exclusively into biomass and citrate synthesis, carbon balances for the three 6-L fermentations were calculated based on theoretical yields (Additional file 3: Table S2). All balances closed within ± 5%—a robust recovery even for a straightforward citric acid fermentation—indicating no appreciable by-product formation [27]. Although carbon-dioxide was not directly measured, net CO_2_ release is inherently minimized because pyruvate-carboxylase-mediated anaplerotic carboxylation fixes the CO_2_ from pyruvate decarboxylation, while growth limitation during the production phase further reduces growth-associated CO_2_ output. The lack of alternative metabolic sinks was further confirmed by the absence of metabolites other than citrate (gluconate, oxalate or polyols) in the culture supernatant analysis, deeming the experimental system appropriate for the objectives of this study.

### Comparative performance of the A. niger double transporter mutant under production conditions

Under low-manganese conditions, all four tested strains achieved final specific molar citrate yields (Y_p/s_) exceeding 80% (Table 2).

**Table 2:** Fermentation performance of *A. niger* parental, single, and double transporter mutant strains grown at 6-L scale bioreactors. For additional kinetic metrics, including final biomass concentrations, maximal productivities, and overall glucose consumption rates, readers are referred to Supplementary Tables S2 and S3

*Aspergillus niger* strain	Molar citric acid yield (%) Fermentation time (h) Final citric acid titers (g L^− 1^)		
	Low Mn	High Mn	Untreated
NRRL 2270 (parental)	80.5 ± 2.3 350 120.8 ± 4.8	31.5 ± 3.4 340 46.2 ± 6	32.0 ± 5.7 350 48 ± 4.5
*ΔdmtA* (Mn-transporter deficient)	81.3 ± 1.8 390 120.83 ± 5.1	67.6 ± 2.9 350 100 ± 11	60.1 ± 2.8 350 89.5 ± 3.8
*cexA* ^*OE*^ (citrate exporter overexpressing)	83.5 ± 1.6 330 125.6 ± 5.4	70.3 ± 3.4 310 104.4 ± 6.5	67.8 ± 3.5 310 101.4 ± 2.9
Δ*dmtA*,* cexA*^*OE*^ (*cexA* overexpressed in a *dmtA*-deficient background)	90.1 ± **1.7** 300 135 ± **3.9**	91.2 ± 1.3 250 136.4 ± 5.1	91.4 ± 2.5 250 136.4 ± 4.5

**Fig. 1:**
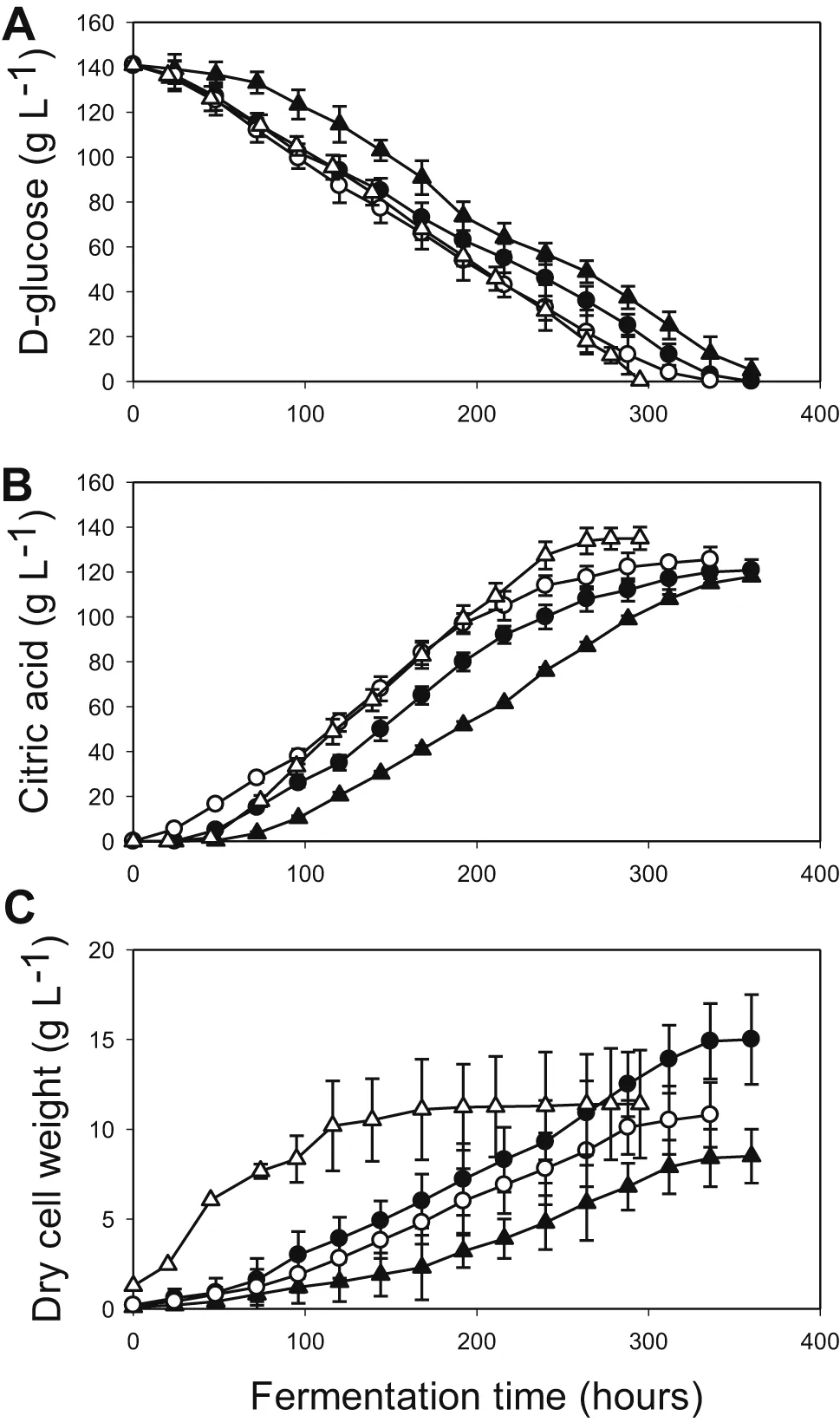
Kinetics of D-glucose utilization (**A**), citric acid production (**B**) and biomass formation (**C**) under production (= low manganese) conditions. The investigated strains are the NRRL 2270 (⬤), JPO_1 (*ΔdmtA*; ▲), CSFG_7012_1 (*cexA*^*OE*^; O), CSFG_7013_1 (*ΔdmtA*, *cexA*^*OE*^; ρ). Fermentations were performed at a 6-L scale in triplicate; error bars represent standard deviations for each measured concentration. In some instances, error bars are smaller than the symbols representing the mean values

While yield performance did not differ significantly between the parental strain and the two single transporter mutants, the double transporter mutant showed a significant increase, reaching 90%. Moreover, statistically significant differences were observed in production rates, which correlated positively with fermentation duration (Fig. 1B). The Δ*dmtA* mutant required 390 h to reach a final titer comparable to those achieved by the *cexA*^*OE*^ and parental strains in 330 h and 350 h, respectively (Table 2). Accordingly, the Δ*dmtA* mutant accumulated citrate significantly slower (maximum production rate: 0.60 g L^− 1^ h^− 1^, whereas the *cexA*^*OE*^ and double transporter mutants outpaced the parental strain NRRL2270 (0.66 and 0.74 g L^− 1^ h^− 1^ vs. 0.62 g L^− 1^ h^− 1^, respectively; Additional file 4: Table S3). Qualitatively similar trends were observed in glucose consumption; the Δ*dmtA* strain exhibited the significantly slowest carbon uptake, while the other two mutants showed the fastest rates with nearly identical profiles (Fig. 1A). Although the parental strain’s uptake remained intermediate, no statistically significant differences were found between the parental, *cexA*^*OE*^ and double transporter strains in this regard.

Comparing dry cell weight (Fig. 1C) and citric acid (Fig. 1B) profiles revealed a kinetic shift in the double transporter mutant. Unlike the other strains, which produced biomass and citric acid simultaneously throughout, the double mutant showed biphasic kinetics: it reached maximum biomass within 100 h, with no increase thereafter while shifting exclusively to citric acid production. In contrast, the other three strains continued to show incremental growth until the end of the process (Fig. 1C). The biphasic kinetic shift in the double-transporter mutant reflects a growth-decoupled production profile, a metabolic engineering paradigm achieved by pairing metabolic blocks with exporter overexpression ([28]). Initially, available carbon resources support biomass accumulation. However, once intracellular citrate reaches a threshold, the overexpressed CexA exporters rapidly deplete the internal pool. This active efflux relieves metabolic feedback inhibition, diverting the carbon flux away from biomass synthesis and locking the cells into an exclusive production phase. Consequently, the double mutant transitions into a strict production profile, whereas the parental strain maintains incremental growth.

While the slower growth of the Δ*dmtA* mutant relative to the parental strain was previously attributed to the lack of the high-affinity manganese transporter [16], the double transporter mutant—carrying the same *dmtA* deletion—surpassed the Δ*dmtA* mutant strain in growth rate during the first 100 h. Although *A. niger* possesses alternative manganese transporters [29, 30] that ensure viability under manganese-limited conditions, the mechanism by which the simultaneous overexpression of *cexA* apparently induces these systems remains to be elucidated.

## Comparative performance of the A. niger double transporter mutant under high manganese conditions

**Fig. 2:**
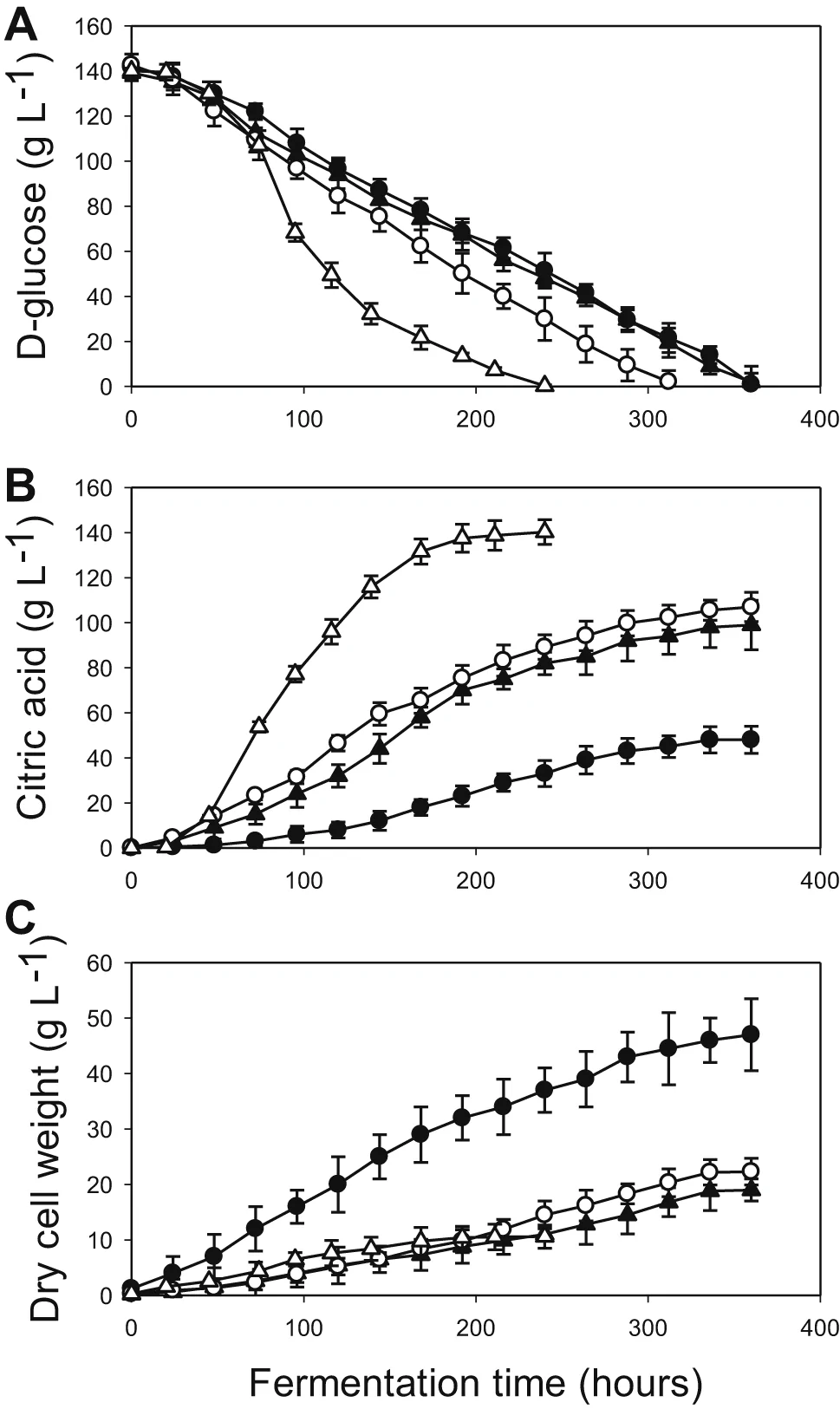
Kinetics of D-glucose utilization (**A**), citric acid production (**B**) and biomass formation (**C**) under high manganese conditions. The investigated strains are the NRRL 2270 (⬤), *ΔdmtA* (▲), *cexA*^*OE*^ (O), *ΔdmtA*; *cexA*^*OE*^ (ρ). Fermentations were performed at a 6-L scale in triplicate; error bars represent standard deviations for each measured concentration. In some instances, error bars are smaller than the symbols representing the mean values

While standard fungal media typically contain 0.1 to 10 mg L^− 1^ of manganese to support normal metabolism [31], the 100 µg L^− 1^ “high” manganese concentration used here lies at the lower limit of this physiological range. Despite the persistent phosphate and nitrogen limitations in the growth medium, the NRRL2270 strain produced approximately 46 g L^− 1^ of biomass (DCW) from the initial 140 g L^− 1^ glucose (Y_x/s_ = 0.33), which is comparable to its citrate yield of approximately 46 g L^− 1^. (Additional file 3: Table S2; Fig. 2B). In contrast, all three transporter mutants yielded significantly higher citrate titers with concomitantly reduced biomass, maintaining an overflow metabolism even under “high manganese” conditions (Table 2). The double mutant vastly outperformed both single mutants; its molar citric acid yield exceeded that achieved under “low manganese” conditions, while its fermentation period was significantly compressed, from 300 to 250 h (Table 2). This efficiency was driven by rapid carbon uptake: the maximal and the overall glucose consumption rate (1.61 and 0.56 g L^− 1^ h^− 1^, respectively) exceeded that of the second fastest strain, the *cexA*^*OE*^ mutant, by 178 and 22% (Fig. 2A; Additional file 4: Table S3). Although the double mutant exhibited accelerated biomass production during the first 150 h, growth subsequently ceased—again highlighting the biphasic nature of the fermentation with this strain—resulting in the lowest final DCW among all strains (9.1 g L^− 1^; Fig. 2C). This metabolic shift directly accounts for the superior citrate yield. Furthermore, the shortened fermentation time implies a reduced maintenance energy requirement, additionally contributing to the performance of this strain.

In general, untreated 6-L scale cultures with initial manganese concentrations between 148 and 165 µg L^− 1^ behaved similarly to those maintained under high-manganese conditions (Table 2). Specific molar yields trended higher for all strains; however, these variations were statistically insignificant. This outcome aligns with expectations. As among the four first-row transition metal ions—manganese, iron (Fe^2+^), copper (Cu^2+^) and zinc (Zn^2+^)—crucial to fungal physiology and organic acid overflow, only manganese is documented to suppress high-yield citric acid fermentation [32]. Indeed, the inhibitory effects frequently attributed to iron [33] are likely artifactual, stemming from manganese impurities within commercial iron salts that can reach up to 0.5% (w/w).

Beyond substrate-derived contamination, large-scale industrial stainless-steel bioreactors pose a significant corrosion risk under hyper-acidic manufacturing conditions (pH < 2). Since these steel alloys may contain up to 2% manganese [23], corroded vessel walls can leach the metal into the medium [14]. To evaluate this phenomenon, the double transporter mutant was cultivated in a 15-L stainless-steel fermenter using untreated medium to simulate worst-case industrial manganese loads. Despite these elevated levels, the double mutant achieved molar citric acid yields identical to those obtained in 6-L glass bioreactors with ion-exchanged media.

To mitigate the manganese sensitivity of citric acid production, several medium-treatment methods are employed such as cation exchange [34], ferrocyanide precipitation [35], and the addition of copper ions to counteract manganese toxicity [36]. These approaches are costly and labor-intensive. By contrast, the double transporter mutant mitigated both the adverse effect of elevated manganese content of the untreated medium and metal leaching from stainless steel components at low pH [14]. Furthermore, this resilience eliminates the need for electrochemical polishing required to prevent reactor-wall leaching. Maintaining high citric acid productivity without prior manganese depletion therefore offers significant techno-economic advantages.

### The morphology of the double transporter mutant is shaped by dmtA deletion

Manganese concentration alters *A. niger* morphology and citric acid yield [37, 38]; even minor fluctuations trigger a pellet-to-filamentous transition that decreases production [39]. Under manganese deficiency, the mycelium forms swollen hyphae and compact, pellet-like aggregates with a smooth surface due to altered branching and impaired cell wall synthesis, as manganese acts as a cofactor for morphogenesis enzymes [37]. Conversely, manganese sufficiency promotes hyphal elongation and dispersed filamentous growth; this morphology increases broth viscosity, impairing mixing and mass transfer characteristics in the bioreactor [38, 40, 41].

We previously demonstrated that DmtA activity influences morphology [16]. The *ΔdmtA* strain exhibits a production-optimized morphology even when cultivated in a medium containing a high (100 µg L^− 1^) manganese concentration. This is because the morphology is dictated by the intracellular manganese levels—in which DmtA plays a crucial regulatory role—rather than the nominal manganese content of the external medium [16]. The same phenomenon was observed in the *ΔdmtA*/*cexA*^*OE*^ double mutant: the culture exhibited a pellet-dominated morphology across both low and high manganese concentrations, as well as under untreated conditions (~ 160 µg L^− 1^ manganese), during both the rapid growth phase (24 h) and the late stationary phase (240 h). Morphological characterization across a 10–100 µg L^− 1^ manganese gradient in 500-mL shake-flasks revealed a shift in morphology around 70 µg L^− 1^, manifested by smaller, less compact pellets with sparse hairy peripheries and vacuolated, globose cells (Additional file 5: Figure S2). These observations suggest that the morphology of the double transporter mutant is predominantly influenced by the lack of DmtA, although potential synergistic effects with *cexA* overexpression remain to be fully elucidated. A similar dominance of manganese availability over other medium parameters in controlling morphology has recently been observed in *A. terreus* itaconate fermentation [42]. This parallel supports the view that manganese and its uptake acts as a primary morphological regulator across organic acid-producing *Aspergillus* platforms, whereas transport capacity modulate metabolic output rather than hyphal architecture.

### Performance of the A. niger double transporter mutant in 150 L scale-up fermentation

Scaling a laboratory bioprocess (0.5–10 L) to industrial manufacturing (20,000–2,000,000 L) requires a pilot-plant intermediary (100–10,000 L) to model full-scale dynamics [43]. Unlike lab systems, industrial bioreactors exhibit environmental non-uniformity [44], creating pH, substrate, temperature and dissolved oxygen gradients that impair process performance [45].

**Fig. 3:**
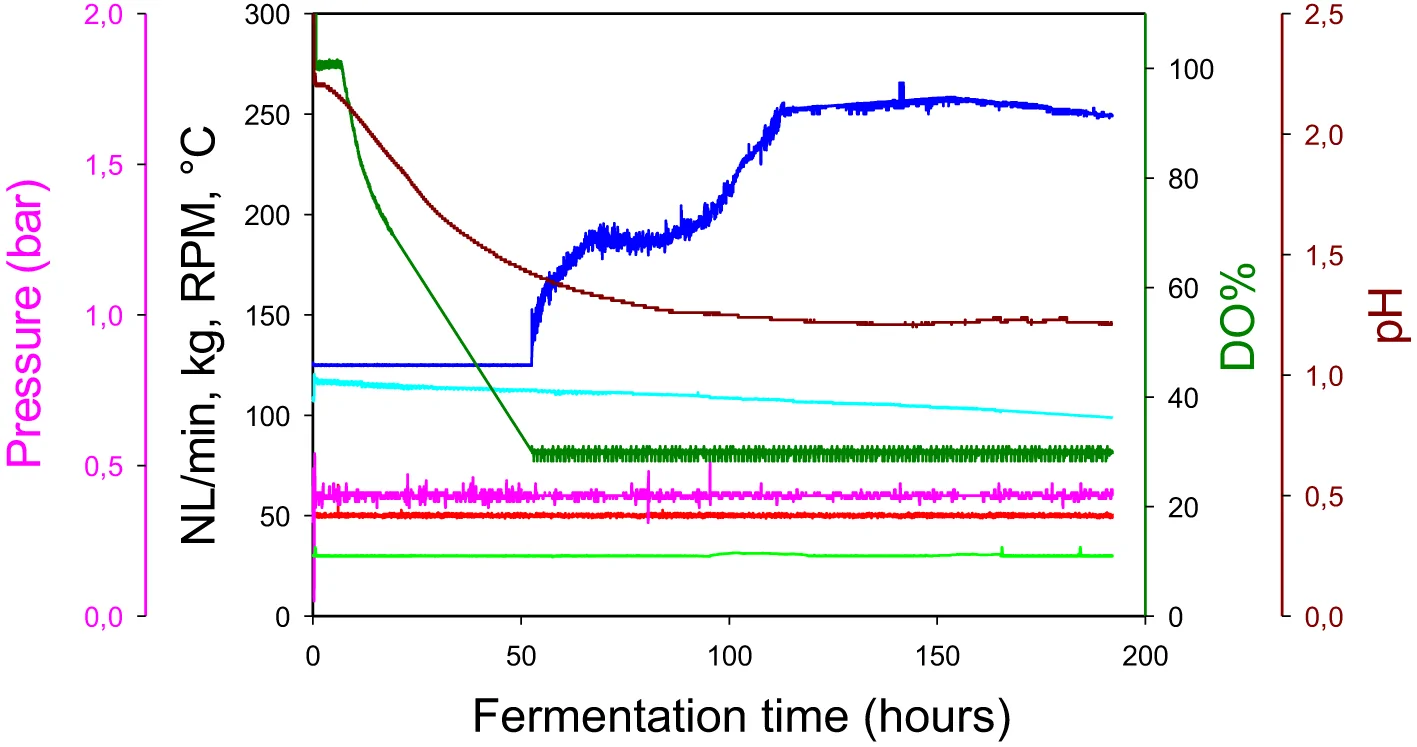
Kinetic profile of citric acid fermentation by the *ΔdmtA ΔglaA::cexA* mutant strain using untreated medium in a 150-L pilot-scale bioreactor. Pressure (pink), aeration (red), temperature (light green), and dissolved oxygen (DO, dark green) were actively controlled, while pH (dark red) and vessel weight (light blue) were monitored. Upon reaching a 30% threshold, the DO level was maintained by automated adjustment of the agitation speed (blue). Process control and data acquisition were managed via Yokogawa Centum CS300 software, interfaced with a Programmable Logic Controller (PLC) for real-time regulation. The PLC maintained process parameters at designated setpoints through automated valve actuation in response to sensor feedback

Critical to scale-up is the translation of process parameters [46]. Highly oxygen-demanding citric acid fermentations require effective mixing to ensure adequate oxygen transfer [47], making initial agitation speed critical due to its direct proportionality to shear force [48]. Because identical impeller types were used across all fermenter scales, initial agitation speeds were calculated directly from impeller diameters and tip speeds [49]. Agitation control maintained DO at 30% throughout fermentation (Fig. 3), while pH remained uncontrolled, mirroring laboratory-scale conditions. Airflow was scaled to 50 NL/min based on the vessel volume per minute (VVM) method [49]. Unlike laboratory setups, pilot-scale operation required monitoring vessel weight to compensate for batch evaporation losses and regulating pressure. While laboratory overpressure is minimal and driven solely by aeration to prevent contamination, the pilot bioreactor was maintained at a 0.4 bar overpressure. This introduced a novel environmental stressor for the microorganism, compounded by increased hydrostatic pressure from the higher liquid column [50].

**Fig. 4:**
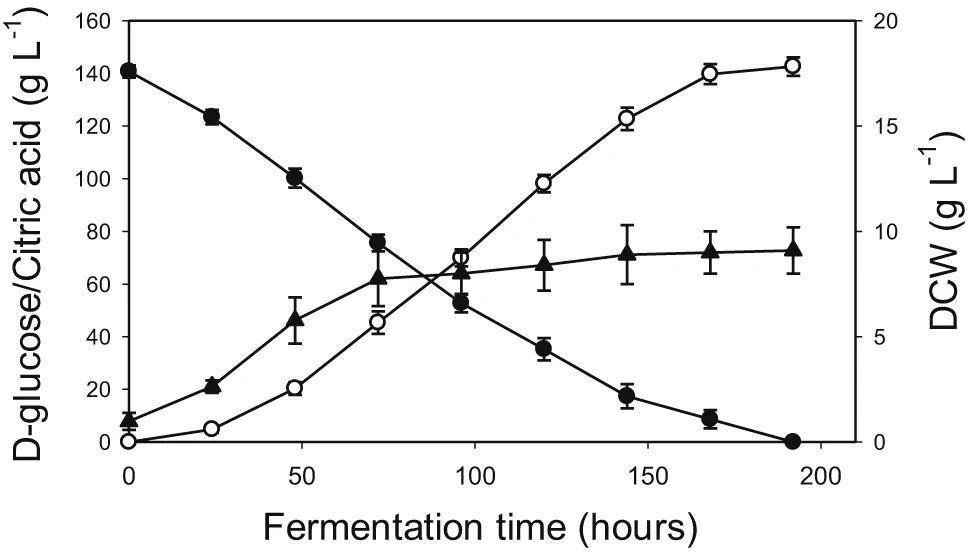
Kinetics of D-glucose utilization (⬤), citric acid production (O) and biomass formation (▲) of *ΔdmtA*; *cexA*^*OE*^ at untreated conditions. Fermentations were performed at a 150-L scale pilot bioreactor in triplicate; error bars represent standard deviations for each measured concentration. In some instances, error bars are smaller than the symbols representing the mean values

Fermentations using the double mutant in non-ion-exchanged medium yielded specific molar citric acid yields above 0.9 at both the 6-L and 15-L scales, representing some of the highest values reported in the literature. Furthermore, applying the aforementioned process technology to the pilot-scale fermentation resulted in a specific molar yield of 0.91 (Fig. 4). The fermentation retained its biphasic profile, characterized by rapid initial biomass formation followed by intensive citric acid accumulation (Fig. 4).

Cell morphology exhibited the same characteristic overflow-associated forms observed at the 6-L scale. However, the fermentation period was significantly shortened, with the culture completely consuming the initial 140 g L^− 1^ glucose within 192 h. This reduction offers substantial cost savings given the high operational expenses of industrial-scale citric acid fermenters. Taken together, these scale-up validation trials demonstrate that integrating the double transporter mutant with an optimized bioprocess technology provides a robust and economically viable framework for next-generation industrial citric acid production.

## Conclusions

By overcoming the critical citrate transport bottleneck and eliminating high-affinity manganese uptake, this study presents a novel *Aspergillus niger* cell factory maintaining reproducible citric acid productivity and consistent morphology across all experimental generations, thereby confirming its long-term industrial robustness. This double transporter mutant strain ensures high-yield citrate accumulation while suppressing biomass formation even under conditions of manganese abundance. Its scalability up to 150-L bioreactors demonstrates potential for cost-effective, unpurified industrial-scale citric acid manufacturing that simplifies process logistics and enhances sustainability.

## Supplementary Information

Supplementary Material 1. Figure S1. Bioreactor configurations used for citric acid fermentation and scale-up. (A) 6 L glass fermenter; (B) 15 L stainless steel fermenters; (C) 150 L stainless steel pilot-scale fermenter. Table S1: Manganese concentration (µg L^− 1^) in tap water across different campuses of the University of Debrecen. The three campuses are located in distinct parts of the city and supplied by different water treatment plants. Data were collected over a 1.5-year period. Fermentations were conducted at the Egyetem Square campus. Table S2. Carbon balances of *Aspergillus niger* NRRL2270, single-, and double-transporter mutant cultures across three initial manganese concentrations. Fermentations were performed in 6-L bioreactors under optimized citrate-producing conditions using 140 g L^− 1^ D-glucose as the sole carbon source. Theoretical calculations were based on mean maximum concentrations of biomass (DCW) and citrate empirically determined from triplicate fermentations. For clarity, standard deviations are omitted. Detailed calculation methodologies (molar yields, carbon requirements, and balances) are provided in Materials and Methods. Table S3. Derived kinetic parameters of *Aspergillus niger* fermentations for the NRRL2270, single, and double mutant strains. Parameters were calculated from dry cell weight (DCW), residual glucose concentration, and citric acid titer measurements. Data are presented as a function of initial manganese levels in the growth medium at a 6-L scale, alongside pilot-scale evaluation of the double mutant at a 150-L scale. Figure S2. Morphological characterization of the *Aspergillus niger* mutant strain CSFG_7013_1 (*ΔdmtA/cexA*^*OE*^) across a 10–100 µg L^-1^ manganese gradient. Micrographs represent samples grown in 100 mL Erlenmeyer flasks at 48 h.

## Data Availability

No datasets were generated or analysed during the current study.
