# Supplementary material for: Eliminating manganese sensitivity and boosting citric acid production in Aspergillus niger through transporters manipulation
Source: Microb Cell Fact. 2026 Aug 6;25:188. doi: 10.1186/s12934-026-03082-y (PMC13602490; doi:10.1186/s12934-026-03082-y)
Supplement: Supplementary file 1 [file 12934_2026_3082_MOESM1_ESM.pdf]

Supplementary Material

Eliminating manganese sensitivity and boosting citric acid production in *Aspergillus niger* through transporters manipulation

Alexandra Márton[1], Josee Vandal[2], Vivien Bíró[1#], Erzsébet Fekete[1*], Erzsébet Sándor[3], Béla Kovács[3], Adrian Tsang[2], and Levente Karaffa[1]

[1]Department of Biochemical Engineering, Faculty of Science and Technology, University of Debrecen, Debrecen, Hungary

[2]Centre for Structural and Functional Genomics, Concordia University, Montreal, QC, Canada

[3]Institute of Plant Protection, Faculty of Agricultural and Food Science and Environmental Management, University of Debrecen, Debrecen, Hungary

#Present address: Department of Molecular Biotechnology and Microbiology, Faculty of Science and Technology, University of Debrecen, Debrecen, Hungary

* Corresponding author

Tel.: +36 52 512 900 ext. 62488

E-mail: kicsizsoka@yahoo.com

Additional file 1: Figure S1: Bioreactor configurations used for citric acid fermentation and scale-up.

Additional file 2: Table S1: Manganese concentration (µg L^{-1}) in tap water across different campuses of the University of Debrecen.

Additional file 3: Table S2: Carbon balances of *Aspergillus niger* wild-type, single-, and double-transporter mutant cultures across three initial manganese concentrations.

Additional file 4: Table S3: Derived kinetic parameters of *Aspergillus niger* fermentations for the wild-type, single, and double mutant strains.

Additional file 5: Figure S2: Morphological characterization of the *Aspergillus niger* mutant strain CSFG_7013_1 (ΔdmtA/cexAOE) across a 10-100 µg L^{-1} manganese gradient

Figure S1. Bioreactor configurations used for citric acid fermentation and scale-up. (A) 6 L glass fermenter; (B) 15 L stainless steel fermenters; (C) 150 L stainless steel pilot-scale fermenter.

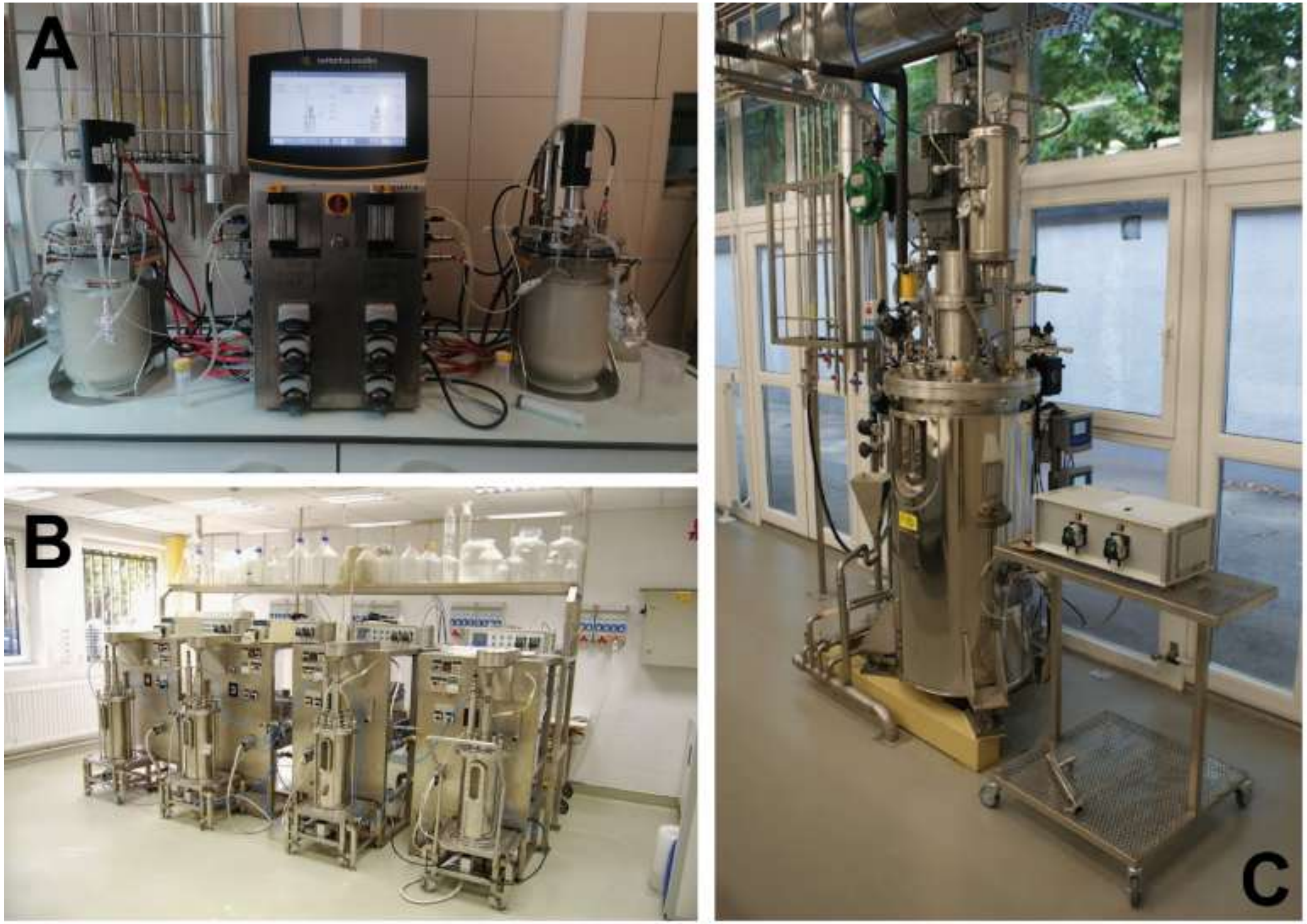

Supplementary Table S1: Manganese concentration (µg L^{-1}) in tap water across different campuses of the University of Debrecen. The three campuses are located in distinct parts of the city and supplied by different water treatment plants. Data were collected over a 1.5-year period. Fermentations were conducted at the Egyetem Square campus.

**Sampling location: Debrecen, Egyetem square 1. (University of Debrecen, Chemistry Building)**												
	**January**	**February**	**March**	**April**	**May**	**June**	**July**	**August**	**September**	**October**	**November**	**December**
**2024**	-	-	-	-	-	-	-	23.4	23.1	20.4	18.8	19.2
**2025**	9.1	9	13.7	8.4	11.1	14.4	12.3	8.1	14.4	10.5	12.3	8.6

**Sampling location: Debrecen, Böszörményi street 138. (University of Debrecen, “A” Building)**												
	**January**	**February**	**March**	**April**	**May**	**June**	**July**	**August**	**September**	**October**	**November**	**December**
**2024**	-	-	-	-	-	-	-	19.6	5.2	5.1	5	4.8
**2025**	14.2	15.8	20.7	14.8	6.4	5.4	6.2	8.4	7.3	7.5	10.4	6.7

**Sampling location: Debrecen, Kassai street 26. (University of Debrecen, Campus Hotel)**												
	**January**	**February**	**March**	**April**	**May**	**June**	**July**	**August**	**September**	**October**	**November**	**December**
**2024**	-	-	-	-	-	-	-	2.5	5.2	5.5	5.1	5.6
**2025**	5.3	5.2	5.1	5	5.5	5.2	6.4	5.6	5.7	5.1	5.6	5.6

Supplementary Table S2. Carbon balances of *Aspergillus niger* wild-type, single-, and double-transporter mutant cultures across three initial manganese concentrations. Fermentations were performed in 6-L bioreactors under optimized citrate-producing conditions using 140 g L^{-1} D-glucose as the sole carbon source. Theoretical calculations were based on mean maximum concentrations of biomass (DCW) and citrate empirically determined from triplicate fermentations. For clarity, standard deviations are omitted. Detailed calculation methodologies (molar yields, carbon requirements, and balances) are provided in Materials and Methods.

Low manganese conditions

**Initial D-glucose: 140 g/L**	**Strains**			
	**WT**	***ΔdmtA* mutant**	***cexA***	**double mutant**
$DCW_{max.}$ (g/L)	15	8.8	10.8	9
Biomass yield (%)	11	6.3	7.7	6.5
$Citrate_{max.}$(g/L)	120.8	120.83	125.6	135
Molar citrate yield (mol/mol)	0.8	0.81	0.84	0.9
Theoretical carbon requirement for DCW (g/L)	13.33	7.82	9.6	8
Theoretical carbon requirement for citrate (g/L)	45.3	45.31	47.1	50.62
Overall theoretical carbon requirement (DCW + citrate; g/L)	58.6	53.13	56.7	58.62
Carbon balance, % (theoretical/consumed)	**105**	**95**	**101**	**105**

High manganese conditions

Initial D-glucose: 140 g/L	Strains			
	**WT**	**Δ*dmtA* mutant**	***cexA^{OE}* mutant**	**double mutant**
$DCW_{max.}$ (g/L)	46	19	22	9.1
Biomass yield (%)	33	13.6	15.7	6.5
$Citrate_{max.}$(g/L)	46.2	100	104.4	136.4
Molar citrate yield (mol/mol)	0.31	0.67	0.7	0.91
Theoretical carbon requirement for DCW (g/L)	40.88	16.88	19.55	8.08
Theoretical carbon requirement for citrate (g/L)	17.3	37.5	39.2	51.15
Overall theoretical carbon requirement (DCW + citrate; g/L)	58.2	54.4	58.7	59.23
Carbon balance, % (theoretical/consumed)	**104**	**97.1**	**105**	**105**

Untreated conditions

**Initial D-glucose: 140 g/L**	**Strains**			
	**WT**	***ΔdmtA* mutant**	***cexA^{OE}* mutant**	**double mutant**
$DCW_{max.}$ (g/L)	46	22	23	9
Biomass yield (%)	33	15.7	16.4	6.4
$Citrate_{max.}$(g/L)	48	89.5	101.4	136.4
Molar citrate yield (mol/mol)	0.32	0.6	0.68	0.91
Theoretical carbon requirement for DCW (g/L)	40.88	19.55	20.4	8
Theoretical carbon requirement for citrate (g/L)	18	33.56	38	51.15
Overall theoretical carbon requirement (DCW + citrate; g/L)	58.88	53.11	58.4	59.15
Carbon balance, % (theoretical/consumed)	**105**	**95**	**104**	**105**

Supplementary Table S3. Derived kinetic parameters of *Aspergillus niger* fermentations for the wild-type, single, and double mutant strains. Parameters were calculated from dry cell weight (DCW), residual glucose concentration, and citric acid titer measurements. Data are presented as a function of initial manganese levels in the growth medium at a 6-L scale, alongside pilot-scale evaluation of the double mutant at a 150-L scale.

	**NRRL 2270 (6 L)**			***ΔdmtA* (6 L)**			***cexA*^{OE} (6 L)**			***ΔdmtA/cexA*^{OE} (6 L)**			***ΔdmtA/cexA*^{OE} (150 L)**
	Low Mn^{2+}	High Mn^{2+}	Untreated	Low Mn^{2+}	High Mn^{2+}	Untreated	Low Mn^{2+}	High Mn^{2+}	Untreated	Low Mn^{2+}	High Mn^{2+}	Untreated	Untreated
**Maximal glucose consumption rate (g L^{-1} h^{-1})**	0.54	0.58	0.57	0.71	0.64	0.66	0.57	0.58	0.6	0.59	1.61	0.76	1.02
**Overall glucose consumption rate (g L^{-1} h^{-1})**	0.4	0.41	0.4	0.34	0.39	0.38	0.43	0.46	0.47	0.47	0.56	0.49	0.73
**Maximal productivity (g citrate L^{-1} h^{-1})**	0.625	0.25	0.24	0.6	0.58	0.58	0.66	0.625	0.64	0.74	1.36	0.87	1.17
**Maximal biomass production rate (g DCW L^{-1} h^{-1})**	0.067	0.21	0.22	0.046	0.096	0.1	0.042	0.087	0.08	0.15	0.088	0.12	0.13
**Maximal specific growth rate (h^{-1})**	0.007	0.022	0.023	0.008	0.006	0.007	0.009	0.008	0.008	0.038	0.036	0.036	0.039

Figure S2. Morphological characterization of the *Aspergillus niger* mutant strain CSFG_7013_1 (*ΔdmtA/cexA*^{OE}) across a 10-100 μg L^{-1} manganese gradient. Micrographs represent samples grown in 100 mL Erlenmeyer flasks at 48 h.

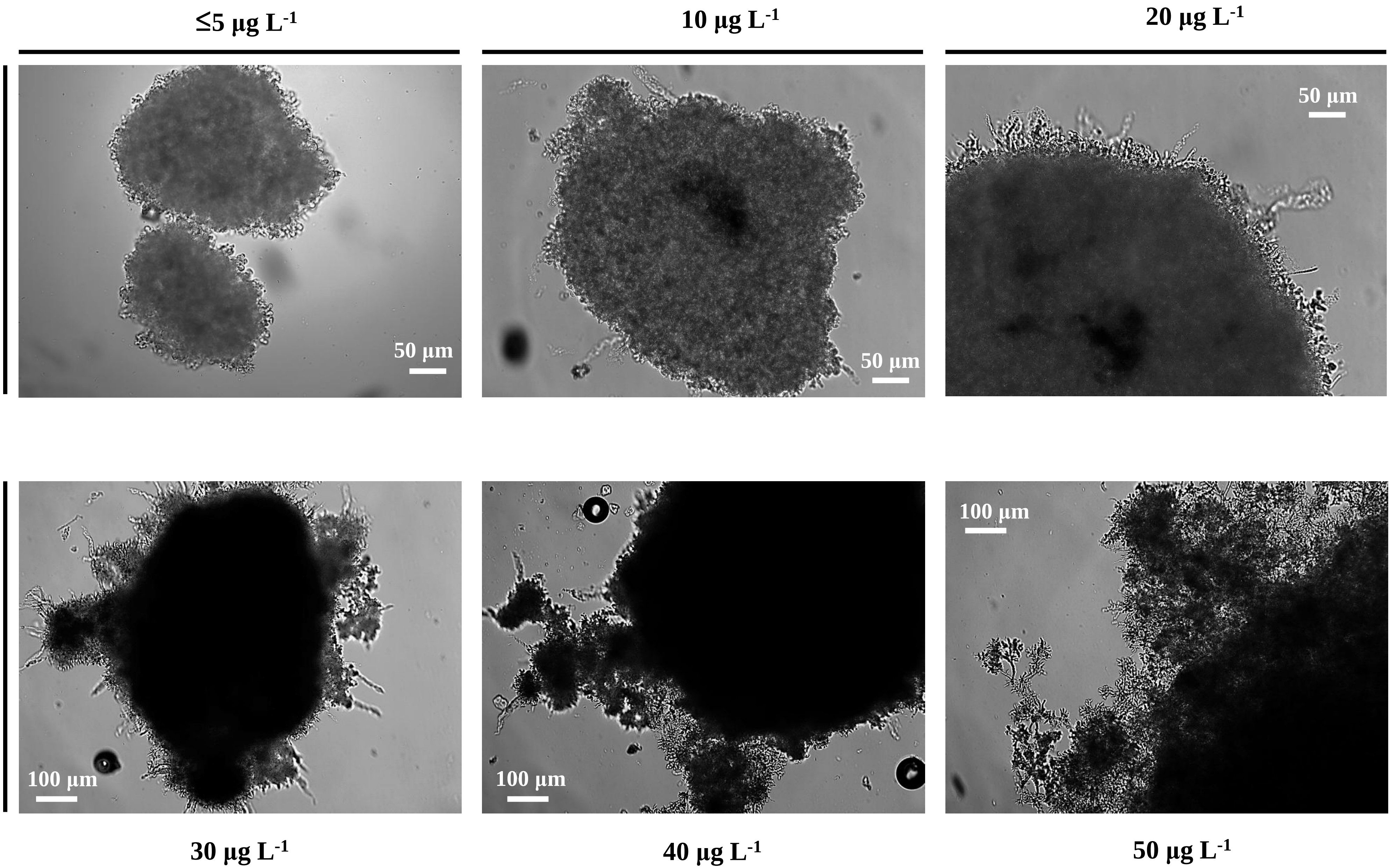

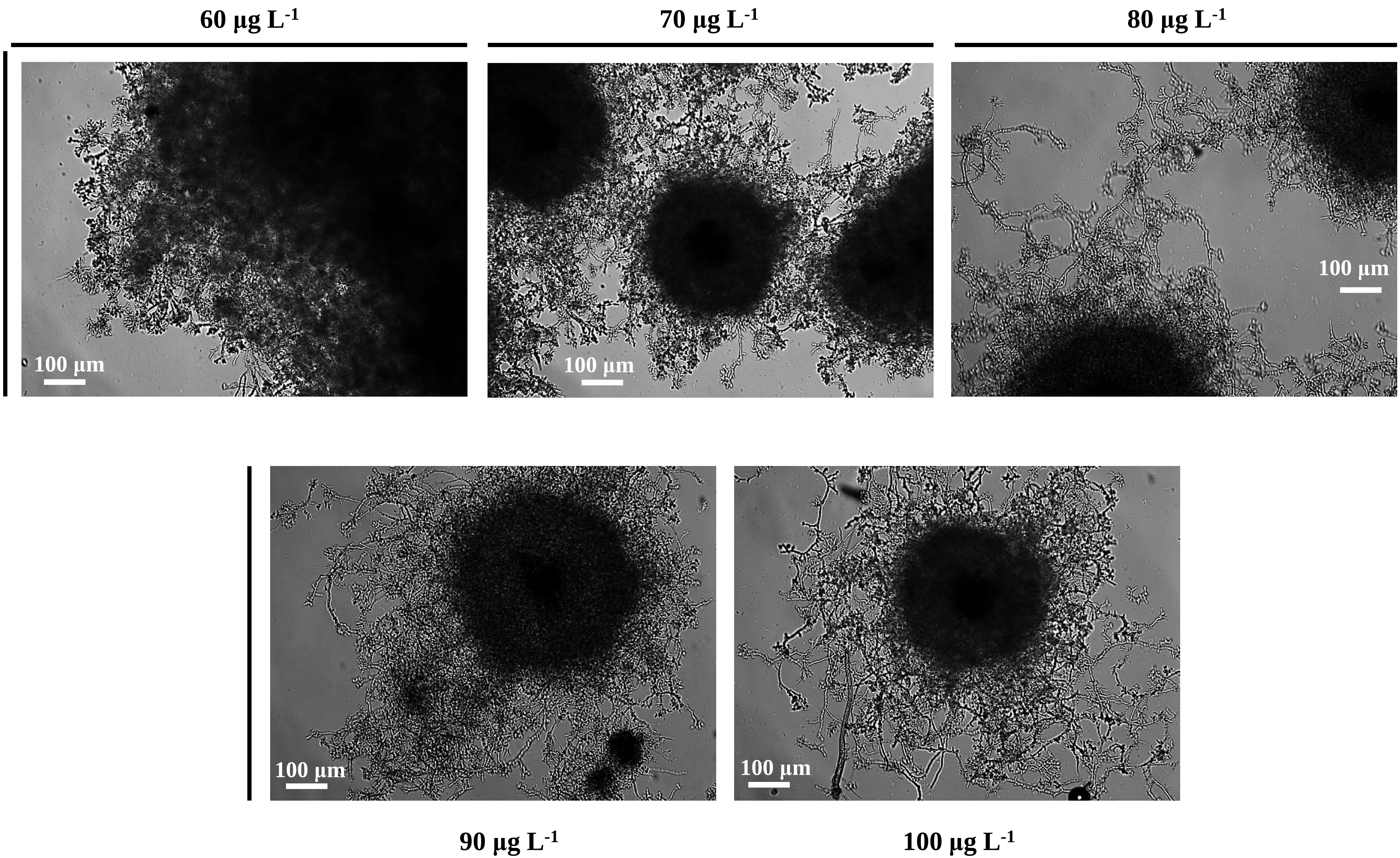
60 µg L-1
70 µg L-1
80 µg L-1
100 µm
100 µm
100 µm
100 µm
100 µm
90 µg L-1
100 µg L-1
